# Sixty Years of Progress in Medicine and Surgery

**Published:** 1910-05

**Authors:** 


### Sixty Years of Progress in Medicine and Surgery

**Dr. Abraham Jacobi's Eightieth Birthday will be Officially Celebrated May 6, 1910, by the Medical Profession—Preparations to Celebrate His Eightieth Birthday with Honors—He has Seen Many Improvements in Practice.**

[*New York Tribune*, April 3, 1910.]

FROM leeching and blood letting to blood transfusion, from the suffocation treatment of pneumonia to the open air method, from surgery a barbaric torture to the marvels of painless operation, from slitting children's throats to inserting a tube in the windpipe, from the doctrine that physicians should chiefly care to make good autopsies to the belief that they should save lives, from pathological guesswork to the germ theory of disease —all this has been seen and lived through by a New York physician, who, at eighty years, is still busily practising his profession.

Dr. Abraham Jacobi is not merely old and vigorous. He is distinguished. He has been not a passive spectator but an active participant in the medical evolution and revolution of the last sixty years. He is considered the chief American authority on children's diseases and has done eminent work in other fields. An official celebration of his eightieth birthday on May 6 will be conducted by the Medical Society of the State of New York, and doubtless the entire country will take a hand in the celebration. A bronze bust of Dr. Jacobi, made by Joseph Davidson, will be placed in Mount Sinai Hospital, with which he has been connected for half a century, and the library in that institution will be named after him. A new children's ward in the German Hospital, endowed by Mrs. Anna Woerishoffer to the extent of $100,000, will be known as the Abraham Jacobi children's division. There will be dinners, addresses, resolutions.

It is suspected that in putting on his bays Dr. Jacobi will give them a slightly rakish tilt and crack a witticism. It is his way when anything personal to him comes up. Broadness of view rather than mere modesty is the cause of this attitude. He regards individual knowledge and achievement as comparatively small. He does not believe in the great man theory, and considers hero worship to be one of the diseases of infancy. Not that he lacks appreciation of youthful naivete. He likes young people and keeps himself young by associating with them. His recreation is playing with his grandchildren, four little McAnenys, whose papa is borough president of Manhattan.

Dr. Jacobi and the McAnenys live together in a big house at No. 19 East 47th street. It may be recalled that Dr. Mary Putnam Jacobi, wife of the physician and herself a practitioner of note, died in 1906. She was the first graduate woman pharmacist in the United States and the first woman to graduate from the Paris Medical University.

When the writer called at the Jacobi house the other morning the waiting rooms were crowded with denizens of the East side and of Fifth avenue. East siders who were treated as babies by Dr. Jacobi half a century ago now bring their grand-children to him for treatment. Three assistant physicians and a trained nurse are needed to take care of the heterogenous callers. The veteran physician had begun work at 8 o'clock in the morning, and did not get through until 2 p. m., after which he might have been persuaded to make a professional call in Brooklyn or to attend a dinner in the evening. It was not until after office hours that Dr. Jacobi would see the interviewer.

A short, little man, with a leonine head of profuse, wavy, gray hair, stood in a spacious back room of the old-fashioned house amid bookcases filled with medical works and journals and surmounted by numbers of marble busts. It would appear that marble busts and the reading of detective stories, especially those of Gaboriau, are mild hobbies of Dr. Jacobi. The physician of eighty asked the visitor to take a seat, but he himself stood and remained standing for a considerable time, while he advanced playful, ingenious and correct arguments why he should not break his practice of not talking for publication. He pleaded that age was no merit, that he did not wish to set a bad example to younger members of the medical profession, and that the evolution of medicine in his lifetime had been better summarised by others than he could do it.

It was a reference to the revolution of 1848 and the late death of Dr. Jacobi's jail colleague in that historic event—Frederick Lessner, who carried the manuscript of the memorable Communist Manifesto to the printer—which checkmated the arguments against publicity. For Dr. Jacobi is an ardent social physician, who believes that a healthy society would not produce many diseased individuals. He was punished for holding this supposedly dangerous and unpatriotic view with a term of eighteen months in a German prison. This was in 1851, when he had just attained his majority and had been graduated from the University of Bonn. It was a sort of post-graduate course for the young doctor and also his first confinement case. Frederick Lessner at the same time got six years in jail. When Dr. Jacobi learned not long ago that Lessner, a man of eighty-five, was living in poverty in London, he sent him a sum of money.

I believe still in the socialist ideal, said Dr. Jacobi, with a smile and a voice of quiet conviction, but I am today an evolutionist rather than a revolutionist. Society, of course, will not always do what we wish. I may desire a peaceful development and it may occur otherwise. What must be will be,—with emphasis the last words were spoken. . . . When I came here in 1853—in a sailing ship—I had to walk a mile and a half from my lodgings in New York to put a letter in the mailbox. The postoffice since then has become a great socialistic institution. So with the public schools and the public health departments. Everything is moving in one direction. Some day we shall arrive.

When I began the study of medicine in 1847 autopsies were considered by many physicians to be the main thing and their ambition was to find out how the patient died, not to cure him. The Vienna school of medicine was in vogue and its high priests considered that the ideal patient was the one who was satisfied to be examined before death by Skoda and after death by Rokitansky. A well-known professor of medicine, Dietl, said that "the physician should be judged by the extent of his knowledge and not by the extent of his cures. Our power is in knowledge, not in deeds."

There was certainly a scarcity of deeds, whether or not there was a plethora of knowledge. Various systems flourished, like that of Brown, of England, who thought that every disease was the result of excitement or depression and gave drugs accordingly. Then there was the homeopathic doctrine that the less the medicine the greater its power, and drugs were administered in fifty-thousandth dilution of their strength.

All apothecaries kept leeches and a doctor always had a lancet in his vest pocket so that he could bleed patients with expedition. To let blood was the golden rule in the treatment of inflammatory diseases. It is well-known that Cavour, the Italian statesman, was bled to death by his physicians in 1861. No one dared to open windows and give fresh air to patients in feverish diseases. Oxygen was regarded as poison for victims of pneumonia. Cold water was given sparingly as a drink and in its place the nastiest hot drinks, like camomile tea, were administered.

Surgery was performed without anesthetics until, in 1847, Dr. Morton, an American, discovered ether. This anesthetic was introduced as a proprietary article, but the profession compelled the inventor to give up the secret and the monopoly of an indispensable drug. Opium had been used to stupify the patient in place of a complete anesthetic. I saw many operations without anesthetics. I operated in 1851 with chloroform in a clinical test examination.

Of course the major operations common today, abdominal and so forth, were impossible without anesthesia, for no patient could have lived under the shock and pain, not to mention the lack of absolute quiescence necessary in fine operations. It was bad enough to have a leg taken off while consciousness remained. In order to minimise pain the oldtime surgeon tried to work with lightning speed. My old teacher, Langenbeck, was going to amputate a leg and an Englishman was on hand to see the operation. As the surgeon grasped the knife the Englishman took out his spectacles and started to put them on so as to watch the operation, but before the spectacles were on the Englishman's nose the leg was off. Today not speed but safety is required in operations. Asepsis and the use of antiseptics—surgical cleanliness—have been big factors in the marvelous development of surgery.

Before O'Dwyer, one of the few patient Americans, invented his windpipe tube for croup and diphtheria thousands of children had their throats slit open in the operation of tracheotomy. A European physician told me that this terrible operation used to unnerve him completely. He performed it without anesthetic.

The germ theory of disease, with its great consequence in antiseptics, and the discovery of antitoxin for many plagues dates from about 1870, and the great pioneers were Pasteur and Koch. After Pasteur had made his studies in fermentation Koch found the germ of tuberculosis, then of cholera and has since done a great deal for the increase of our knowledge, started by the English, of sleeping sickness. Bacteriological laboratory work is now obligatory for medical students.

The great increase of knowledge has made it necessary to extend the time of study in medical colleges. Half a century ago two years was considered sufficient. Now we have four years, with much longer courses and more subjects taught. We have added to thoroughness also, and the so-called didactic lecturer is less esteemed than formerly. Bedside and clinical work and practical laboratory work are now regarded as of the greatest importance. We are still defective in bedside instruction."

In a preface to a lately published eight-volume collection of Dr. Jacobi's essays, addresses, scientific papers and other writings, edited by Dr. William J. Robinson, the veteran physician says:

The first of my professional successes was the fact that it took my first patient only a fortnight after my new shingle began to ornament No. 20 Howard street to call on me with his 25-cent fee. That was in November, 1853. I must have had quite a reputation at that time, for his only excuse for coming at all was that he had heard of me. I think I must have gathered many more such fees, for after less than four years I was one of the founders of the German dispensary, in which treatment was strictly gratuitous. . . .

After seventeen years I scored quite a success, when I—refusing to resign—got myself expelled from a public institution for proving a 100 per cent. mortality among the babies there, and for insisting upon a farming out system.

Dr. Jacobi still doesn't believe in keeping foundlings in hospitals, except for the treatment of diseases. For one thing, infantile epidemics sweep through an institution, and for another no scientific care in wholesale fashion compensates for the individual mother love obtained by farming out the youngsters among private families. A baby given out to a poor Italian mother at $2.00 or $3.00 a week, with a little supervision and instruction of the foster mother, he thinks thrives better than if it were kept in the best conducted institution.

Speaking of professional ethics and abuses, Dr. Jacobi remarks:

But that there are men in the profession who give or demand bribes, take commissions from apothecaries, instrument and bandage makers, nurses—men and women—manufacturers, in the shape of cash or stock, consultants both medical and surgical—that is no longer professional, no longer even the competition of an honest tradesman. It is robbery, which pollutes the moral atmosphere of professional life and fleeces the consumer of your services—that is, the patient.

The unprecedented honor of the offer of a chair in the University of Berlin—a professorship of the diseases of children—came to Dr. Jacobi about fifteen years ago. No American had ever before been invited to such a place. Dr. Jacobi was regularly chairman of the American section of physicians at the International Medical Congresses held every three years in various capitals of Europe.

I smoke one cigar a day and I drink a little Rhine wine," said the octogenarian physician when asked about his daily regimen.

There is value in the proper use of alcohol, both for sick and well. In severe cases of diphtheria alcohol counteracts the poison in the system, and an enormous quantity of it can be given to a child without causing intoxication and with beneficial results. I am a moderate eater, not a vegetarian.

Dr. Jacobi contemplates writing his memoirs this summer during his vacation with the big and little McAnenys at the family cottage at Lake George.

